# Supplementary material for: Determination of drought tolerance of different strawberry genotypes
Source: PeerJ. 2023 Feb 28;11:e14972. doi: 10.7717/peerj.14972 (PMC9983431; doi:10.7717/peerj.14972)
Supplement: Supplemental Information 1 [file peerj-11-14972-s001.pdf]

Abbreviations

CWSI: Crop water stress index

Dap: Day after planting

E: Transpiration rate

Eo: Cumulative free surface water evaporation from Class A pan at irrigation interval

Epan: Pan's ecaporation

FAO: The Food and Agriculture Organization of the United Nations

gs: Stomatal conductance

IR: Irrigation

IRT: Infrared thermometer

IWUE: Irrigation water use efficiency

Kcp: Crop-pan coefficients

LL: Non-water-stressed baseline

P: Wetted area

Par: Photosynthetic available radiation

Pn: Net photosynthesis

Ta = Air temperature

Tc: Canopy temperature

UL: Non-transpiring upper baseline

VPD: Vapor pressure deficit

WS: Water stress

WW: Well-watered
