# Supplementary material for: Determination of drought tolerance of different strawberry genotypes
Source: PeerJ. 2023 Feb 28;11:e14972. doi: 10.7717/peerj.14972 (PMC9983431; doi:10.7717/peerj.14972)
Supplement: Supplemental Information 2 [file peerj-11-14972-s002.pdf]

In [39]:
```
import numpy as np
import pandas as pd
import matplotlib
pd.set_option('display.max_rows', 5)
```

In [43]:
```
from bokeh.plotting import figure, show
from bokeh.io import output_notebook
import bokeh
from bokeh.models import SingleIntervalTicker, LinearAxis
from bokeh.models import  ColumnDataSource,Range1d, LabelSet, Label

from bokeh.resources import INLINE
import bokeh.io
bokeh.io.output_notebook(INLINE)
from sklearn.linear_model import LinearRegression, LogisticRegression
```

Loading BokehJS ...

In [80]:
```
loc='../cwsixiwue2.xlsx'
main_df=pd.read_excel(loc)
ww_df = main_df[main_df.irrigation == 'WW']
ws_df = main_df[main_df.irrigation == 'WS']
main_df[main_df.genotypes=='R'].IWUE.values
```

Out[80]:
```
array([1.25390456, 1.06936556, 1.2451662 , 1.29568246, 1.89455622,
       1.28245672])
```

In [ ]:
```
from bokeh.models import ColumnDataSource, Label, LabelSet, Range1d
from bokeh.plotting import figure, output_file, show
ww_df['genotypes']=ww_df.apply(lambda x: str(x.genotypes),axis=1)
ws_df['genotypes']=ws_df.apply(lambda x: str(x.genotypes),axis=1)
source = ColumnDataSource(data=dict(CWSI=ww_df.CWSI.values,
                                    IWUE=ww_df.IWUE.values,
                                    genotypes=ww_df.genotypes.values))
source2 = ColumnDataSource(data=dict(CWSI=ws_df.CWSI.values,
                                    IWUE=ws_df.IWUE.values,
                                    genotypes=ws_df.genotypes.values))
```

Adding Correlation Lines and numbers

In [73]:
```
from numpy.polynomial.polynomial import polyfit

b_ww, m_ww = polyfit(ww_df.CWSI.values, ww_df.IWUE.values, 1)
b_ws, m_ws = polyfit(ws_df.CWSI.values, ws_df.IWUE.values, 1)
```

In [91]:
```
fig=figure(width=1000,height=300,x_axis_type=None,y_range=(1,2.05))
fig.scatter(ww_df.CWSI.values,ww_df.IWUE.values,size=15,color='blue',alpha=0
fig.scatter(ws_df.CWSI.values,ws_df.IWUE.values,size=15,color='crimson',alph
# fig.xaxis.ticker.num_minor_ticks = 30
ticker = SingleIntervalTicker(interval=0.02, num_minor_ticks=10)
xaxis = LinearAxis(ticker=ticker)
fig.add_layout(xaxis, 'below')

fig.xaxis.axis_label='CWSI'
fig.yaxis.axis_label='IWUE (g/mm)'

labels = LabelSet(x='CWSI', y='IWUE', text='genotypes', level='glyph',text_f
              text_color='black',x_offset=10, y_offset=10, source=source, re
labels2 = LabelSet(x='CWSI', y='IWUE', text='genotypes', level='glyph',text_
```

```
            text_color='black',x_offset=10, y_offset=10, source=source2, r

fig.line(ww_df.CWSI.values,ww_df.CWSI.values*m_ww+b_ww,line_width=2,color='b
fig.line(ws_df.CWSI.values,ws_df.CWSI.values*m_ws+b_ws,line_width=2,color='b
fig.add_layout(labels)
fig.add_layout(labels2)
fig.xaxis.axis_label_text_font_size = "15pt"
fig.yaxis.axis_label_text_font_size = "15pt"
fig.axis.axis_label_text_font_style = 'bold'
fig.legend.label_text_font_size = "15pt"
fig.yaxis.major_label_text_font_size = "15pt"
fig.xaxis.major_label_text_font_size = "15pt"

show(fig)
```

In [92]:

```
display(ww_df.corr())
display(ws_df.corr())
```

	CWSI	IWUE	YIELD
**CWSI**	1.000000	-0.799112	-0.799112
**IWUE**	-0.799112	1.000000	1.000000
**YIELD**	-0.799112	1.000000	1.000000

	CWSI	IWUE	YIELD
**CWSI**	1.000000	-0.656658	-0.656658
**IWUE**	-0.656658	1.000000	1.000000
**YIELD**	-0.656658	1.000000	1.000000
