# Supplementary material for: Determination of drought tolerance of different strawberry genotypes
Source: PeerJ. 2023 Feb 28;11:e14972. doi: 10.7717/peerj.14972 (PMC9983431; doi:10.7717/peerj.14972)
Supplement: Supplemental Information 3 [file peerj-11-14972-s003.pdf]

In [1]:
```
from bokeh.plotting import figure, show
from bokeh.io import output_notebook
import bokeh
from bokeh.models import SingleIntervalTicker, LinearAxis
from bokeh.models import  ColumnDataSource,Range1d, LabelSet, Label

from bokeh.resources import INLINE
import bokeh.io
bokeh.io.output_notebook(INLINE)
from sklearn.linear_model import LinearRegression, LogisticRegression
```

Loading BokehJS ...

In [8]:
```
import pandas as pd
df_in=pd.read_excel('cwsi graphs.xlsx')
df_in
```

Out[8]:

	dap (day after planting)	Rubygem_WW	Rubygem_WS	Festival_WW	Festival_WS	33_WW	33_WS
**0**	187	0.453410	0.512142	0.463439	0.568373	0.433272	0.536314
**1**	200	0.466236	0.562847	0.483620	0.606649	0.458194	0.563546
**2**	214	0.494854	0.602851	0.503632	0.626767	0.492502	0.610687
**3**	228	0.502097	0.655298	0.511455	0.625715	0.502574	0.618856
**4**	242	0.514993	0.681962	0.528596	0.648729	0.523381	0.668177
**5**	256	0.508686	0.699196	0.547685	0.705053	0.522456	0.687126
**6**	271	0.521590	0.713184	0.562728	0.735635	0.531753	0.738184

In [47]:
```
import re
s=figure(width=1000,height=300,x_axis_type=None)
keys=[a for a in df_in.keys() if '_' in a]
markers=['square','square','circle','circle','triangle','triangle','diamond'
for key,count,marker in zip(keys,range(len(keys)),markers):
    if re.search('WS',key):
        if count<3:
            s.line(df_in['dap (day after planting)'].values,df_in[key],color
        else:
            s.line(df_in['dap (day after planting)'].values,df_in[key],color
        s.scatter(df_in['dap (day after planting)'].values,df_in[key],color=
    else:
        if count<3:
            s.line(df_in['dap (day after planting)'].values,df_in[key],color
        else:
            s.line(df_in['dap (day after planting)'].values,df_in[key],color
        s.scatter(df_in['dap (day after planting)'].values,df_in[key],color=

ticker = SingleIntervalTicker(interval=10, num_minor_ticks=5)
xaxis = LinearAxis(ticker=ticker)
s.add_layout(xaxis, 'below')
s.add_layout(s.legend[0], 'right')
s.xaxis.axis_label='Day After Planting (DAP)'
s.yaxis.axis_label='CWSI'
s.xaxis.axis_label_text_font_size = "15pt"
s.yaxis.axis_label_text_font_size = "15pt"
s.axis.axis_label_text_font_style = 'bold'
```

```
s.legend.label_text_font_size = "15pt"
s.yaxis.major_label_text_font_size = "15pt"
s.xaxis.major_label_text_font_size = "15pt"

show(s)
```
