# Supplementary material for: Determination of drought tolerance of different strawberry genotypes
Source: PeerJ. 2023 Feb 28;11:e14972. doi: 10.7717/peerj.14972 (PMC9983431; doi:10.7717/peerj.14972)
Supplement: Supplemental Information 7 [file peerj-11-14972-s007.docx]

**Least Squares Fit**

**Response Yield**

**Analysis of Variance**

| Source | DF | Sum of Squares | Mean Square | F Ratio |
| --- | --- | --- | --- | --- |
| Model | 11 | 882504,49 | 80227,7 | 9,2110 |
| Error | 12 | 104519,61 | 8710,0 | Prob > F |
| C. Total | 23 | 987024,10 |  | 0,0003 |

Effect Tests

| Source | Nparm | DF | Sum of Squares | F Ratio | Prob > F |  |
| --- | --- | --- | --- | --- | --- | --- |
| replicate | 2 | 2 | 6181,96 | 0,3549 | 0,7084 |  |
| irrigation | 1 | 1 | 720966,91 | 82,7749 | <.0001 |  |
| irrigation*replicate | 2 | 2 | 11174,62 | 0,6415 | 0,5436 |  |
| Genotype | 3 | 3 | 92547,41 | 3,5418 | 0,0481 |  |
| irrigation*Genotype | 3 | 3 | 51633,59 | 1,9760 | 0,1714 |  |

Effect Details

irrigation

LSMeans Differences Student's t

Alpha=

0,050 t=

2,17881

| Level |  |  | Least Sq Mean |
| --- | --- | --- | --- |
| 100 | A |  | 952,39111 |
| 50 |  | B | 605,74843 |

Levels not connected by same letter are significantly different

Genotype

LSMeans Differences Student's t

Alpha=

0,050 t=

2,17881

| Level |  |  | Least Sq Mean |
| --- | --- | --- | --- |
| 59 | A |  | 866,50534 |
| 33 | A | B | 798,32729 |
| Rubygem | A | B | 755,02364 |
| FESTİVAL |  | B | 696,42280 |

Levels not connected by same letter are significantly different

irrigation*Genotype

LSMeans Differences Student's t

Alpha=

0,050 t=

2,17881

| Level |  |  |  |  | Least Sq Mean |
| --- | --- | --- | --- | --- | --- |
| 100,59 | A |  |  |  | 1067,4209 |
| 100,33 | A | B |  |  | 1029,4283 |
| 100,Rubygem |  | B | C |  | 864,9057 |
| 100,FESTİVAL |  |  | C |  | 847,8096 |
| 50,59 |  |  |  | D | 665,5898 |
| 50,Rubygem |  |  |  | D | 645,1416 |
| 50,33 |  |  |  | D | 567,2263 |
| 50,FESTİVAL |  |  |  | D | 545,0360 |

Levels not connected by same letter are significantly different

**Response IWUE**

**Analysis of Variance**

| Source | DF | Sum of Squares | Mean Square | F Ratio |
| --- | --- | --- | --- | --- |
| Model | 11 | 0,51935320 | 0,047214 | 1,3010 |
| Error | 12 | 0,43548753 | 0,036291 | Prob > F |
| C. Total | 23 | 0,95484073 |  | 0,3282 |

Effect Tests

| Source | Nparm | DF | Sum of Squares | F Ratio | Prob > F |  |
| --- | --- | --- | --- | --- | --- | --- |
| replicate | 2 | 2 | 0,03775253 | 0,5201 | 0,6072 |  |
| irrigation | 1 | 1 | 0,04867293 | 1,3412 | 0,2694 |  |
| irrigation*replicate | 2 | 2 | 0,05362020 | 0,7388 | 0,4982 |  |
| Genotype | 3 | 3 | 0,25466988 | 2,3392 | 0,1250 |  |
| irrigation*Genotype | 3 | 3 | 0,12463766 | 1,1448 | 0,3706 |  |

Effect Details

irrigation

LSMeans Differences Student's t

Alpha=

0,050 t=

2,17881

| Level |  | Least Sq Mean |
| --- | --- | --- |
| 50 | A | 1,3998623 |
| 100 | A | 1,3097948 |

Levels not connected by same letter are significantly different

Genotype

LSMeans Differences Student's t

Alpha=

0,050 t=

2,17881

| Level |  |  | Least Sq Mean |
| --- | --- | --- | --- |
| 59 | A |  | 1,5030727 |
| 33 | A | B | 1,3632904 |
| Rubygem | A | B | 1,3401886 |
| FESTİVAL |  | B | 1,2127626 |

Levels not connected by same letter are significantly different

irrigation*Genotype

LSMeans Differences Student's t

Alpha=

0,050 t=

2,17881

| Level |  |  | Least Sq Mean |
| --- | --- | --- | --- |
| 50,59 | A |  | 1,5381535 |
| 50,Rubygem | A | B | 1,4908985 |
| 100,59 | A | B | 1,4679918 |
| 100,33 | A | B | 1,4157417 |
| 50,33 | A | B | 1,3108391 |
| 50,FESTİVAL | A | B | 1,2595582 |
| 100,Rubygem |  | B | 1,1894788 |
| 100,FESTİVAL |  | B | 1,1659670 |

Levels not connected by same letter are significantly different
