# Supplementary material for: Determination of drought tolerance of different strawberry genotypes
Source: PeerJ. 2023 Feb 28;11:e14972. doi: 10.7717/peerj.14972 (PMC9983431; doi:10.7717/peerj.14972)
Supplement: Supplemental Information 8 [file peerj-11-14972-s008.docx]

**Least Squares Fit**

**Response Par**

**Analysis of Variance**

| Source | DF | Sum of Squares | Mean Square | F Ratio |
| --- | --- | --- | --- | --- |
| Model | 25 | 4719280,3 | 188771 | 1255,296 |
| Error | 46 | 6917,5 | 150 | Prob > F |
| C. Total | 71 | 4726197,8 |  | <.0001 |

**Effect Tests**

| Source | Nparm | DF | Sum of Squares | F Ratio | Prob > F |  |
| --- | --- | --- | --- | --- | --- | --- |
| Period | 2 | 2 | 3641356,0 | 12107,2 | <.0001 |  |
| Genotype | 3 | 3 | 18626,8 | 41,2883 | <.0001 |  |
| Genotype  *Period | 6 | 6 | 11232,0 | 12,4484 | <.0001 |  |
| irrigation | 1 | 1 | 874282,7 | 5813,83 | <.0001 |  |
| irrigation*Period | 2 | 2 | 169305,9 | 562,9274 | <.0001 |  |
| irrigation*Genotype | 3 | 3 | 2602,9 | 5,7697 | 0,0020 |  |
| irrigation*Genotype  *Period | 6 | 6 | 1645,5 | 1,8237 | 0,1153 |  |
| replicate | 2 | 2 | 228,5 | 0,7598 | 0,4735 |  |

**Effect Details**

**Period**

**LSMeans Differences Student's t**

Alpha=

0,050 t=

2,0129

| Level |  |  |  | Least Sq Mean |
| --- | --- | --- | --- | --- |
| b | A |  |  | 618,45833 |
| c |  | B |  | 608,41667 |
| a |  |  | C | 136,45833 |

Levels not connected by same letter are significantly different

**Genotype**

**LSMeans Differences Student's t**

Alpha=

0,050 t=

2,0129

| Level |  |  |  |  | Least Sq Mean |
| --- | --- | --- | --- | --- | --- |
| 59 | A |  |  |  | 479,44444 |
| 33 |  | B |  |  | 456,38889 |
| Rubygem |  |  | C |  | 445,61111 |
| FESTİVAL |  |  |  | D | 436,33333 |

Levels not connected by same letter are significantly different

**Genotype*Period**

**LSMeans Differences Student's t**

Alpha=

0,050 t=

2,0129

| Level |  |  |  |  |  |  | Least Sq Mean |
| --- | --- | --- | --- | --- | --- | --- | --- |
| 59,b | A |  |  |  |  |  | 647,50000 |
| 59,c | A |  |  |  |  |  | 640,33333 |
| 33,b | A | B |  |  |  |  | 633,83333 |
| 33,c |  | B |  |  |  |  | 620,00000 |
| Rubygem,b |  |  | C |  |  |  | 603,00000 |
| Rubygem,c |  |  | C | D |  |  | 590,50000 |
| FESTİVAL,b |  |  | C | D |  |  | 589,50000 |
| FESTİVAL,c |  |  |  | D |  |  | 582,83333 |
| 59,a |  |  |  |  | E |  | 150,50000 |
| Rubygem,a |  |  |  |  | E |  | 143,33333 |
| FESTİVAL,a |  |  |  |  | E |  | 136,66667 |
| 33,a |  |  |  |  |  | F | 115,33333 |

Levels not connected by same letter are significantly different

**irrigation**

**LSMeans Differences Student's t**

Alpha=

0,050 t=

2,0129

| Level |  |  | Least Sq Mean |
| --- | --- | --- | --- |
| 100 | A |  | 564,63889 |
| 50 |  | B | 344,25000 |

Levels not connected by same letter are significantly different

**irrigation*Period**

**LSMeans Differences Student's t**

Alpha=

0,050 t=

2,0129

| Level |  |  |  |  |  | Least Sq Mean |
| --- | --- | --- | --- | --- | --- | --- |
| 100,b | A |  |  |  |  | 762,00000 |
| 100,c | A |  |  |  |  | 753,83333 |
| 50,b |  | B |  |  |  | 474,91667 |
| 50,c |  |  | C |  |  | 463,00000 |
| 100,a |  |  |  | D |  | 178,08333 |
| 50,a |  |  |  |  | E | 94,83333 |

Levels not connected by same letter are significantly different

**irrigation*Genotype**

**Least Squares Means Table**

| Level | Least Sq Mean |  | Std Error |
| --- | --- | --- | --- |
| 100,33 | 568,00000 |  | 4,0876485 |
| 100,59 | 597,11111 |  | 4,0876485 |
| 100,FESTİVAL | 537,22222 |  | 4,0876485 |
| 100,Rubygem | 556,22222 |  | 4,0876485 |
| 50,33 | 344,77778 |  | 4,0876485 |
| 50,59 | 361,77778 |  | 4,0876485 |
| 50,FESTİVAL | 335,44444 |  | 4,0876485 |
| 50,Rubygem | 335,00000 |  | 4,0876485 |

**irrigation*Genotype*Period**

**LSMeans Differences Student's t**

Alpha=

0,050 t=

2,0129

| Level |  |  |  |  |  |  |  |  |  |  |  | Least Sq Mean |
| --- | --- | --- | --- | --- | --- | --- | --- | --- | --- | --- | --- | --- |
| 100,59,b | A |  |  |  |  |  |  |  |  |  |  | 796,66667 |
| 100,59,c | A | B |  |  |  |  |  |  |  |  |  | 788,66667 |
| 100,33,b | A | B |  |  |  |  |  |  |  |  |  | 780,33333 |
| 100,33,c |  | B |  |  |  |  |  |  |  |  |  | 773,66667 |
| 100,Rubygem,b |  |  | C |  |  |  |  |  |  |  |  | 744,00000 |
| 100,Rubygem,c |  |  | C | D |  |  |  |  |  |  |  | 733,00000 |
| 100,FESTİVAL,b |  |  | C | D |  |  |  |  |  |  |  | 727,00000 |
| 100,FESTİVAL,c |  |  |  | D |  |  |  |  |  |  |  | 720,00000 |
| 50,59,b |  |  |  |  | E |  |  |  |  |  |  | 498,33333 |
| 50,59,c |  |  |  |  | E |  |  |  |  |  |  | 492,00000 |
| 50,33,b |  |  |  |  | E |  |  |  |  |  |  | 487,33333 |
| 50,33,c |  |  |  |  |  | F |  |  |  |  |  | 466,33333 |
| 50,Rubygem,b |  |  |  |  |  | F | G |  |  |  |  | 462,00000 |
| 50,FESTİVAL,b |  |  |  |  |  | F | G |  |  |  |  | 452,00000 |
| 50,Rubygem,c |  |  |  |  |  | F | G |  |  |  |  | 448,00000 |
| 50,FESTİVAL,c |  |  |  |  |  |  | G |  |  |  |  | 445,66667 |
| 100,59,a |  |  |  |  |  |  |  | H |  |  |  | 206,00000 |
| 100,Rubygem,a |  |  |  |  |  |  |  | H |  |  |  | 191,66667 |
| 100,FESTİVAL,a |  |  |  |  |  |  |  |  | I |  |  | 164,66667 |
| 100,33,a |  |  |  |  |  |  |  |  | I |  |  | 150,00000 |
| 50,FESTİVAL,a |  |  |  |  |  |  |  |  |  | J |  | 108,66667 |
| 50,59,a |  |  |  |  |  |  |  |  |  | J | K | 95,00000 |
| 50,Rubygem,a |  |  |  |  |  |  |  |  |  | J | K | 95,00000 |
| 50,33,a |  |  |  |  |  |  |  |  |  |  | K | 80,66667 |

Levels not connected by same letter are significantly different

**Response Pn**

**Analysis of Variance**

| Source | DF | Sum of Squares | Mean Square | F Ratio |
| --- | --- | --- | --- | --- |
| Model | 25 | 606,24514 | 24,2498 | 159,7807 |
| Error | 46 | 6,98139 | 0,1518 | Prob > F |
| C. Total | 71 | 613,22653 |  | <.0001 |

**Effect Tests**

| Source | Nparm | DF | Sum of Squares | F Ratio | Prob > F |  |
| --- | --- | --- | --- | --- | --- | --- |
| Period | 2 | 2 | 216,55444 | 713,4329 | <.0001 |  |
| Genotype | 3 | 3 | 9,75486 | 21,4248 | <.0001 |  |
| Genotype  *Period | 6 | 6 | 3,01556 | 3,3116 | 0,0086 |  |
| irrigation | 1 | 1 | 312,91681 | 2061,792 | <.0001 |  |
| irrigation*Period | 2 | 2 | 60,44111 | 199,1216 | <.0001 |  |
| irrigation*Genotype | 3 | 3 | 2,00153 | 4,3960 | 0,0084 |  |
| irrigation*Genotype  *Period | 6 | 6 | 1,54889 | 1,7009 | 0,1423 |  |
| replicate | 2 | 2 | 0,01194 | 0,0394 | 0,9614 |  |

**Effect Details**

**Period**

**LSMeans Differences Student's t**

Alpha=

0,050 t=

2,0129

| Level |  |  |  | Least Sq Mean |
| --- | --- | --- | --- | --- |
| b | A |  |  | 10,962500 |
| c |  | B |  | 9,779167 |
| a |  |  | C | 6,837500 |

Levels not connected by same letter are significantly different

**Genotype**

**LSMeans Differences Student's t**

Alpha=

0,050 t=

2,0129

| Level |  |  | Least Sq Mean |
| --- | --- | --- | --- |
| 33 | A |  | 9,5777778 |
| 59 | A |  | 9,5388889 |
| FESTİVAL |  | B | 8,8888889 |
| Rubygem |  | B | 8,7666667 |

Levels not connected by same letter are significantly different

**Genotype*Period**

**LSMeans Differences Student's t**

Alpha=

0,050 t=

2,0129

| Level |  |  |  |  |  |  | Least Sq Mean |
| --- | --- | --- | --- | --- | --- | --- | --- |
| 33,b | A |  |  |  |  |  | 11,450000 |
| 59,b | A |  |  |  |  |  | 11,316667 |
| FESTİVAL,b |  | B |  |  |  |  | 10,583333 |
| 59,c |  | B |  |  |  |  | 10,533333 |
| Rubygem,b |  | B | C |  |  |  | 10,500000 |
| 33,c |  |  | C |  |  |  | 10,050000 |
| FESTİVAL,c |  |  |  | D |  |  | 9,300000 |
| Rubygem,c |  |  |  | D |  |  | 9,233333 |
| 33,a |  |  |  |  | E |  | 7,233333 |
| FESTİVAL,a |  |  |  |  | E | F | 6,783333 |
| 59,a |  |  |  |  |  | F | 6,766667 |
| Rubygem,a |  |  |  |  |  | F | 6,566667 |

Levels not connected by same letter are significantly different

**irrigation**

**LSMeans Differences Student's t**

Alpha=

0,050 t=

2,0129

| Level |  |  | Least Sq Mean |
| --- | --- | --- | --- |
| 100 | A |  | 11,277778 |
| 50 |  | B | 7,108333 |

Levels not connected by same letter are significantly different

**irrigation*Period**

**LSMeans Differences Student's t**

Alpha=

0,050 t=

2,0129

| Level |  |  |  |  |  |  | Least Sq Mean |
| --- | --- | --- | --- | --- | --- | --- | --- |
| 100,b | A |  |  |  |  |  | 13,366667 |
| 100,c |  | B |  |  |  |  | 12,791667 |
| 50,b |  |  | C |  |  |  | 8,558333 |
| 100,a |  |  |  | D |  |  | 7,675000 |
| 50,c |  |  |  |  | E |  | 6,766667 |
| 50,a |  |  |  |  |  | F | 6,000000 |

Levels not connected by same letter are significantly different

**irrigation*Genotype**

**Least Squares Means Table**

| Level | Least Sq Mean |  | Std Error |
| --- | --- | --- | --- |
| 100,33 | 11,933333 |  | 0,12985861 |
| 100,59 | 11,622222 |  | 0,12985861 |
| 100,FESTİVAL | 10,866667 |  | 0,12985861 |
| 100,Rubygem | 10,688889 |  | 0,12985861 |
| 50,33 | 7,222222 |  | 0,12985861 |
| 50,59 | 7,455556 |  | 0,12985861 |
| 50,FESTİVAL | 6,911111 |  | 0,12985861 |
| 50,Rubygem | 6,844444 |  | 0,12985861 |

**irrigation*Genotype*Period**

**LSMeans Differences Student's t**

Alpha=

0,050 t=

2,0129

| Level |  |  |  |  |  |  |  |  |  |  |  |  | Least Sq Mean |
| --- | --- | --- | --- | --- | --- | --- | --- | --- | --- | --- | --- | --- | --- |
| 100,33,b | A |  |  |  |  |  |  |  |  |  |  |  | 13,966667 |
| 100,59,b | A | B |  |  |  |  |  |  |  |  |  |  | 13,800000 |
| 100,33,c | A | B | C |  |  |  |  |  |  |  |  |  | 13,366667 |
| 100,59,c |  | B | C |  |  |  |  |  |  |  |  |  | 13,266667 |
| 100,FESTİVAL,b |  |  | C | D |  |  |  |  |  |  |  |  | 12,933333 |
| 100,Rubygem,b |  |  | C | D | E |  |  |  |  |  |  |  | 12,766667 |
| 100,FESTİVAL,c |  |  |  | D | E |  |  |  |  |  |  |  | 12,366667 |
| 100,Rubygem,c |  |  |  |  | E |  |  |  |  |  |  |  | 12,166667 |
| 50,33,b |  |  |  |  |  | F |  |  |  |  |  |  | 8,933333 |
| 50,59,b |  |  |  |  |  | F | G |  |  |  |  |  | 8,833333 |
| 100,33,a |  |  |  |  |  | F | G |  |  |  |  |  | 8,466667 |
| 50,Rubygem,b |  |  |  |  |  |  | G | H |  |  |  |  | 8,233333 |
| 50,FESTİVAL,b |  |  |  |  |  |  | G | H |  |  |  |  | 8,233333 |
| 50,59,c |  |  |  |  |  |  |  | H | I |  |  |  | 7,800000 |
| 100,59,a |  |  |  |  |  |  |  | H | I |  |  |  | 7,800000 |
| 100,FESTİVAL,a |  |  |  |  |  |  |  |  | I | J |  |  | 7,300000 |
| 100,Rubygem,a |  |  |  |  |  |  |  |  |  | J |  |  | 7,133333 |
| 50,33,c |  |  |  |  |  |  |  |  |  | J | K |  | 6,733333 |
| 50,Rubygem,c |  |  |  |  |  |  |  |  |  |  | K | L | 6,300000 |
| 50,FESTİVAL,a |  |  |  |  |  |  |  |  |  |  | K | L | 6,266667 |
| 50,FESTİVAL,c |  |  |  |  |  |  |  |  |  |  | K | L | 6,233333 |
| 50,33,a |  |  |  |  |  |  |  |  |  |  |  | L | 6,000000 |
| 50,Rubygem,a |  |  |  |  |  |  |  |  |  |  |  | L | 6,000000 |
| 50,59,a |  |  |  |  |  |  |  |  |  |  |  | L | 5,733333 |

Levels not connected by same letter are significantly different

**Response C**

**Analysis of Variance**

| Source | DF | Sum of Squares | Mean Square | F Ratio |
| --- | --- | --- | --- | --- |
| Model | 25 | 2075620,8 | 83024,8 | 1592,102 |
| Error | 46 | 2398,8 | 52,1 | Prob > F |
| C. Total | 71 | 2078019,7 |  | <.0001 |

**Effect Tests**

| Source | Nparm | DF | Sum of Squares | F Ratio | Prob > F |  |
| --- | --- | --- | --- | --- | --- | --- |
| Period | 2 | 2 | 1115240,5 | 10693,04 | <.0001 |  |
| Genotype | 3 | 3 | 20452,5 | 130,7337 | <.0001 |  |
| Genotype  *Period | 6 | 6 | 14587,1 | 46,6210 | <.0001 |  |
| irrigation | 1 | 1 | 682696,1 | 13091,52 | <.0001 |  |
| irrigation*Period | 2 | 2 | 213689,6 | 2048,878 | <.0001 |  |
| irrigation*Genotype | 3 | 3 | 11812,4 | 75,5055 | <.0001 |  |
| irrigation*Genotype  *Period | 6 | 6 | 16976,8 | 54,2583 | <.0001 |  |
| replicate | 2 | 2 | 165,9 | 1,5903 | 0,2149 |  |

**Effect Details**

**Period**

**LSMeans Differences Student's t**

Alpha=

0,050 t=

2,0129

| Level |  |  |  | Least Sq Mean |
| --- | --- | --- | --- | --- |
| b | A |  |  | 519,41667 |
| c |  | B |  | 471,79167 |
| a |  |  | C | 234,83333 |

Levels not connected by same letter are significantly different

**Genotype**

**LSMeans Differences Student's t**

Alpha=

0,050 t=

2,0129

| Level |  |  |  |  | Least Sq Mean |
| --- | --- | --- | --- | --- | --- |
| 33 | A |  |  |  | 430,44444 |
| 59 |  | B |  |  | 413,33333 |
| FESTİVAL |  |  | C |  | 407,55556 |
| Rubygem |  |  |  | D | 383,38889 |

Levels not connected by same letter are significantly different

**irrigation**

**LSMeans Differences Student's t**

Alpha=

0,050 t=

2,0129

| Level |  |  | Least Sq Mean |
| --- | --- | --- | --- |
| 100 | A |  | 506,05556 |
| 50 |  | B | 311,30556 |

Levels not connected by same letter are significantly different

**irrigation*Genotype**

**LSMeans Differences Student's t**

Alpha=

0,050 t=

2,0129

| Level |  |  |  |  |  | Least Sq Mean |
| --- | --- | --- | --- | --- | --- | --- |
| 100,33 | A |  |  |  |  | 533,11111 |
| 100,59 | A |  |  |  |  | 528,55556 |
| 100,FESTİVAL |  | B |  |  |  | 488,77778 |
| 100,Rubygem |  |  | C |  |  | 473,77778 |
| 50,33 |  |  |  | D |  | 327,77778 |
| 50,FESTİVAL |  |  |  | D |  | 326,33333 |
| 50,59 |  |  |  |  | E | 298,11111 |
| 50,Rubygem |  |  |  |  | E | 293,00000 |

Levels not connected by same letter are significantly different

**irrigation*Genotype*Period**

**LSMeans Differences Student's t**

Alpha=

0,050 t=

2,0129

| Level |  |  |  |  |  |  |  |  |  |  |  |  |  |  |  | Least Sq Mean |
| --- | --- | --- | --- | --- | --- | --- | --- | --- | --- | --- | --- | --- | --- | --- | --- | --- |
| 100,33,b | A |  |  |  |  |  |  |  |  |  |  |  |  |  |  | 683,66667 |
| 100,59,b | A | B |  |  |  |  |  |  |  |  |  |  |  |  |  | 673,00000 |
| 100,33,c |  | B |  |  |  |  |  |  |  |  |  |  |  |  |  | 663,66667 |
| 100,59,c |  |  | C |  |  |  |  |  |  |  |  |  |  |  |  | 644,00000 |
| 100,FESTİVAL,b |  |  |  | D |  |  |  |  |  |  |  |  |  |  |  | 611,66667 |
| 100,Rubygem,b |  |  |  | D |  |  |  |  |  |  |  |  |  |  |  | 608,00000 |
| 100,FESTİVAL,c |  |  |  |  | E |  |  |  |  |  |  |  |  |  |  | 582,66667 |
| 100,Rubygem,c |  |  |  |  | E |  |  |  |  |  |  |  |  |  |  | 581,33333 |
| 50,33,b |  |  |  |  |  | F |  |  |  |  |  |  |  |  |  | 446,66667 |
| 50,FESTİVAL,b |  |  |  |  |  |  | G |  |  |  |  |  |  |  |  | 401,00000 |
| 50,Rubygem,b |  |  |  |  |  |  | G |  |  |  |  |  |  |  |  | 396,00000 |
| 50,59,c |  |  |  |  |  |  |  | H |  |  |  |  |  |  |  | 352,33333 |
| 50,FESTİVAL,c |  |  |  |  |  |  |  | H | I |  |  |  |  |  |  | 343,33333 |
| 50,59,b |  |  |  |  |  |  |  |  | I |  |  |  |  |  |  | 335,33333 |
| 50,33,c |  |  |  |  |  |  |  |  |  | J |  |  |  |  |  | 305,00000 |
| 50,Rubygem,c |  |  |  |  |  |  |  |  |  | J |  |  |  |  |  | 302,00000 |
| 100,FESTİVAL,a |  |  |  |  |  |  |  |  |  |  | K |  |  |  |  | 272,00000 |
| 100,59,a |  |  |  |  |  |  |  |  |  |  | K |  |  |  |  | 268,66667 |
| 100,33,a |  |  |  |  |  |  |  |  |  |  |  | L |  |  |  | 252,00000 |
| 50,FESTİVAL,a |  |  |  |  |  |  |  |  |  |  |  |  | M |  |  | 234,66667 |
| 100,Rubygem,a |  |  |  |  |  |  |  |  |  |  |  |  | M |  |  | 232,00000 |
| 50,33,a |  |  |  |  |  |  |  |  |  |  |  |  | M |  |  | 231,66667 |
| 50,59,a |  |  |  |  |  |  |  |  |  |  |  |  |  | N |  | 206,66667 |
| 50,Rubygem,a |  |  |  |  |  |  |  |  |  |  |  |  |  |  | O | 181,00000 |

Levels not connected by same letter are significantly different

**Response E**

**Analysis of Variance**

| Source | DF | Sum of Squares | Mean Square | F Ratio |
| --- | --- | --- | --- | --- |
| Model | 25 | 77,318056 | 3,09272 | 1125,615 |
| Error | 46 | 0,126389 | 0,00275 | Prob > F |
| C. Total | 71 | 77,444444 |  | <.0001 |

**Effect Tests**

| Source | Nparm | DF | Sum of Squares | F Ratio | Prob > F |  |
| --- | --- | --- | --- | --- | --- | --- |
| Period | 2 | 2 | 39,381111 | 7166,497 | <.0001 |  |
| Genotype | 3 | 3 | 0,777778 | 94,3590 | <.0001 |  |
| Genotype  *Period | 6 | 6 | 0,255556 | 15,5018 | <.0001 |  |
| irrigation | 1 | 1 | 29,388889 | 10696,26 | <.0001 |  |
| irrigation*Period | 2 | 2 | 7,414444 | 1349,266 | <.0001 |  |
| irrigation*Genotype | 3 | 3 | 0,060000 | 7,2791 | 0,0004 |  |
| irrigation*Genotype  *Period | 6 | 6 | 0,040000 | 2,4264 | 0,0403 |  |
| replicate | 2 | 2 | 0,000278 | 0,0505 | 0,9508 |  |

**Effect Details**

**Period**

**LSMeans Differences Student's t**

Alpha=

0,050 t=

2,0129

| Level |  |  |  | Least Sq Mean |
| --- | --- | --- | --- | --- |
| b | A |  |  | 3,0250000 |
| c |  | B |  | 2,6416667 |
| a |  |  | C | 1,3000000 |

Levels not connected by same letter are significantly different

**Genotype**

**LSMeans Differences Student's t**

Alpha=

0,050 t=

2,0129

| Level |  |  |  |  | Least Sq Mean |
| --- | --- | --- | --- | --- | --- |
| 59 | A |  |  |  | 2,4444444 |
| 33 |  | B |  |  | 2,4000000 |
| Rubygem |  |  | C |  | 2,2555556 |
| FESTİVAL |  |  |  | D | 2,1888889 |

Levels not connected by same letter are significantly different

**Genotype*Period**

**irrigation**

**Least Squares Means Table**

| Level | Least Sq Mean |  | Std Error | Mean |
| --- | --- | --- | --- | --- |
| 100 | 2,9611111 |  | 0,00873623 | 2,96111 |
| 50 | 1,6833333 |  | 0,00873623 | 1,68333 |

**irrigation*Period**

**LSMeans Differences Student's t**

Alpha=

0,050 t=

2,0129

| Level |  |  |  |  |  |  | Least Sq Mean |
| --- | --- | --- | --- | --- | --- | --- | --- |
| 100,b | A |  |  |  |  |  | 3,7833333 |
| 100,c |  | B |  |  |  |  | 3,6000000 |
| 50,b |  |  | C |  |  |  | 2,2666667 |
| 50,c |  |  |  | D |  |  | 1,6833333 |
| 100,a |  |  |  |  | E |  | 1,5000000 |
| 50,a |  |  |  |  |  | F | 1,1000000 |

Levels not connected by same letter are significantly different

**irrigation*Genotype**

**Least Squares Means Table**

| Level | Least Sq Mean |  | Std Error |
| --- | --- | --- | --- |
| 100,33 | 3,0777778 |  | 0,01747247 |
| 100,59 | 3,1000000 |  | 0,01747247 |
| 100,FESTİVAL | 2,8000000 |  | 0,01747247 |
| 100,Rubygem | 2,8666667 |  | 0,01747247 |
| 50,33 | 1,7222222 |  | 0,01747247 |
| 50,59 | 1,7888889 |  | 0,01747247 |
| 50,FESTİVAL | 1,5777778 |  | 0,01747247 |
| 50,Rubygem | 1,6444444 |  | 0,01747247 |

**irrigation*Genotype*Period**

**LSMeans Differences Student's t**

Alpha=

0,050 t=

2,0129

| Level |  |  |  |  |  |  |  |  |  |  |  |  |  |  | Least Sq Mean |
| --- | --- | --- | --- | --- | --- | --- | --- | --- | --- | --- | --- | --- | --- | --- | --- |
| 100,59,b | A |  |  |  |  |  |  |  |  |  |  |  |  |  | 3,9666667 |
| 100,33,b | A |  |  |  |  |  |  |  |  |  |  |  |  |  | 3,9000000 |
| 100,33,c |  | B |  |  |  |  |  |  |  |  |  |  |  |  | 3,7666667 |
| 100,59,c |  | B |  |  |  |  |  |  |  |  |  |  |  |  | 3,7666667 |
| 100,Rubygem,b |  |  | C |  |  |  |  |  |  |  |  |  |  |  | 3,6666667 |
| 100,FESTİVAL,b |  |  | C |  |  |  |  |  |  |  |  |  |  |  | 3,6000000 |
| 100,Rubygem,c |  |  |  | D |  |  |  |  |  |  |  |  |  |  | 3,4666667 |
| 100,FESTİVAL,c |  |  |  | D |  |  |  |  |  |  |  |  |  |  | 3,4000000 |
| 50,59,b |  |  |  |  | E |  |  |  |  |  |  |  |  |  | 2,4333333 |
| 50,33,b |  |  |  |  | E |  |  |  |  |  |  |  |  |  | 2,4000000 |
| 50,Rubygem,b |  |  |  |  |  | F |  |  |  |  |  |  |  |  | 2,1333333 |
| 50,FESTİVAL,b |  |  |  |  |  | F |  |  |  |  |  |  |  |  | 2,1000000 |
| 50,59,c |  |  |  |  |  |  | G |  |  |  |  |  |  |  | 1,8333333 |
| 50,33,c |  |  |  |  |  |  |  | H |  |  |  |  |  |  | 1,7333333 |
| 50,Rubygem,c |  |  |  |  |  |  |  |  | I |  |  |  |  |  | 1,6333333 |
| 100,33,a |  |  |  |  |  |  |  |  | I | J |  |  |  |  | 1,5666667 |
| 100,59,a |  |  |  |  |  |  |  |  | I | J |  |  |  |  | 1,5666667 |
| 50,FESTİVAL,c |  |  |  |  |  |  |  |  |  | J | K |  |  |  | 1,5333333 |
| 100,Rubygem,a |  |  |  |  |  |  |  |  |  |  | K | L |  |  | 1,4666667 |
| 100,FESTİVAL,a |  |  |  |  |  |  |  |  |  |  |  | L |  |  | 1,4000000 |
| 50,Rubygem,a |  |  |  |  |  |  |  |  |  |  |  |  | M |  | 1,1666667 |
| 50,59,a |  |  |  |  |  |  |  |  |  |  |  |  | M | N | 1,1000000 |
| 50,FESTİVAL,a |  |  |  |  |  |  |  |  |  |  |  |  | M | N | 1,1000000 |
| 50,33,a |  |  |  |  |  |  |  |  |  |  |  |  |  | N | 1,0333333 |

Levels not connected by same letter are significantly different
